# Gender and health social enterprises in Africa: a research agenda

**DOI:** 10.1186/s12939-019-0994-2

**Published:** 2019-06-20

**Authors:** Kevin McKague, Sarah Harrison

**Affiliations:** 1 Cape Breton University, 1250 Grand Lake Road, Sydney, Nova Scotia Canada; 2 Toronto, Canada

**Keywords:** Gender equality, Social enterprise, Community health workers, Africa

## Abstract

**Background:**

Health social enterprises in Africa working with community health workers (CHWs) are growing rapidly but understudied. In particular, gender equality issues related to their work has important public health and equity implications.

**Methods:**

Particularly suited for generating timely findings from reviews at the intersection of overlapping disciplines, we utilized the rapid evidence assessment (REA) methodology to identify key unanswered research questions at the intersection of the fields of gender equality, social enterprises and community health workers. The REA used a series of structured Google Scholar searches, expert interviews and bibliography reviews to identify 57 articles in the academic and grey literatures that met the study inclusion criteria. Articles were thematically coded to identify answers to “*What are the most important research questions about the influence of gender on CHWs working with health social enterprises in Africa?”*

**Results:**

The analysis identified six key unanswered research questions relating to 1) equitable systems and structures; 2) training; 3) leadership development and career enhancement; 4) payment and incentives; 5) partner, household and community support; and 6) performance.

**Conclusion:**

This is the first study of its kind to identify the key unanswered research questions relevant to gender equality in health social enterprises in Africa using community health workers. As such, it sets out a research agenda for this newly emerging but rapidly developing area of research and practice with important public health implications.

## Introduction

This paper was motivated by the increasing interdisciplinary convergence of three Sustainable Development Goals (SDGs): Good Health and Well-Being (SDG 3), Gender Equality (SDG 5), and Partnerships for the Goals (SDG 17, which includes working with the private sector) particularly as the convergence of these goals relates to social enterprises using community health workers (CHWs) in Africa. We chose the convergence of these three SDGs because of the their gender issues are increasingly important to health social enterprises utilizing CHWs in Africa. Although these areas are increasingly overlapping and converging in practice, no scholarly studies have yet explored this particular intersection of knowledge.

After the failure of some large-scale government CHW programs in the 1980s [31], the global pendulum has now swung back towards CHW programs [34] with increasing optimism for their potential to fill gaps in health coverage, contribute to the sustainable development goals and reach the more remote and marginalized communities in developing countries [37]. The resurgence of interest in CHWs has also attracted the interest of non-governmental organizations and social enterprises. For example, BRAC Africa, a non-governmental organization, has trained over 6000 CHWs in Uganda, Liberia, Sierra Leone, and South Sudan to provide MCH care via household visits with CHWs generating income through the sale of over-the-counter medicines and other health products. Living Goods, also a non-profit organization, follows a similar enhanced sales agent model using CHWs in Uganda and Kenya and aims to train 50,000 CHWs by 2021 in partnership with Last Mile Health. BRAC and Living Goods CHWs were found to reduce child mortality by 27% compared to control communities [30]. In Kenya, Access Afya, a social business, has established a network of clinics in informal settlements in Nairobi that use CHWs to effectively deliver services and engage community members [27]. In addition, new start-up organizations such as Healthy Entrepreneurs are working with existing government CHWs in Uganda, Kenya, Tanzania, Ghana, and Congo to equip them as agents who can sell medical goods in underserved areas, complementing their role as providers of basic health information.

The majority of CHWs being engaged by social enterprises in Africa are women; however, understanding how gender impacts CHW activities within social enterprises has not yet been studied. Our REA takes the first step in addressing this gap by reviewing the evidence base to explore what we know and do not know about how gender influences the work of CHWs in health social enterprises in Africa. The next section provides an overview of our three key knowledge domains of interest: gender equality, CHWs, and social enterprise. This is followed by a description of our REA methodology and a presentation of our findings with concluding comments.

### Gender equality

Different from sex, which refers to one’s biological characteristics, gender is the socially constructed roles, responsibilities, rights, expectations, and power relations that society holds around being female or male [19, 23, 39]. Gender equality is the capacity for both women and men to live a life they value; to equally enjoy opportunities, freedoms, and resources. Although gender equality has long been recognized as an essential human right [41], research demonstrates that improved gender equality and women’s economic empowerment has a positive impact on economic growth by enhancing efficiencies and productivity [21]. Addressing gender inequalities is also important for improving the health of populations around the world [20, 44], especially among the poorest and most marginalized [18]. The achievement of greater child health outcomes has been linked with empowerment approaches that address MCH behaviours [23] because of the disproportionate effect that poverty has on the lives of women and children [2].

Donors worldwide are affirming the relationship between greater health outcomes and increased gender equality and women’s empowerment [17, 42] and are increasingly being gender intentional by integrating a gender perspective throughout all of their efforts [20]. Similarly, it is argued that CHW programs will be most impactful when they also seek to understand and consider gender norms, roles, and household decision making [32]. Despite this view however, gender equality remains mostly overlooked in the CHW literature.

### Community health workers

Although the definition of a CHW can vary due to the wide diversity of ways CHWs are organized globally, we define CHWs as volunteers who receive basic primary health training, work in their own communities, and are supported by the health system but not formally a part of it [15, 24]. It is estimated that globally, 70% of CHWs are women [24].

A number of factors have led to a renewed interest in the use of CHWs for improving MCH, including the inability of formal health systems to address the primary health care needs of low-income individuals and the prohibitive costs of training medical staff and building hospitals in low-resource settings [15]. Research suggests that when CHWs are managed and supported effectively, CHW programs can reduce maternal and child mortality when compared to facility-based services alone [6]. However, for CHW programs to be most effective, CHWs need to be respected and embedded in a supportive system that includes proper training, sufficient incentives, supervision, and support from the communities (and organizations or government) in which they work [15, 32]. Although the literature has considered gender issues related to CHWs broadly (Mumtaz, Salway, Waseem, & Umer, 2013; [9]), no studies have yet looked at the relevant gender issues for CHWs working with health social enterprises. This remains an important gap in the field.

### Social Enterprise

We define social enterprises as ‘organizations that have created models for efficiently catering to basic human needs that existing markets and institutions have failed to satisfy’ ([35], p. 241). In general, they combine the creativity, action-orientation, and customer-service focus of entrepreneurship with a primary mission to generate positive social benefit [22, 26, 36]. Social enterprises in the field of health are typically designed and governed so that improvements to the business will deliver improvements in health outcomes [26]. While there have been various uses of social enterprises in the health field outside of low-income settings [33], the focus on CHWs for MCH is a new phenomenon with much of the existing research being based on government or faith-based organizations [24]. One notable exception is a study (although not specific to health social enterprises in Africa) on how gender integration can help to optimize the strategies and operations of social enterprises to improve both their business and social impact [10].

Overall, the literature on the impact of gender on health social enterprises in Africa using CHWs is still emerging, with more focused and specific research direction needed. Our REA is designed to address this gap.

## Methodology

**Fig. 1:**
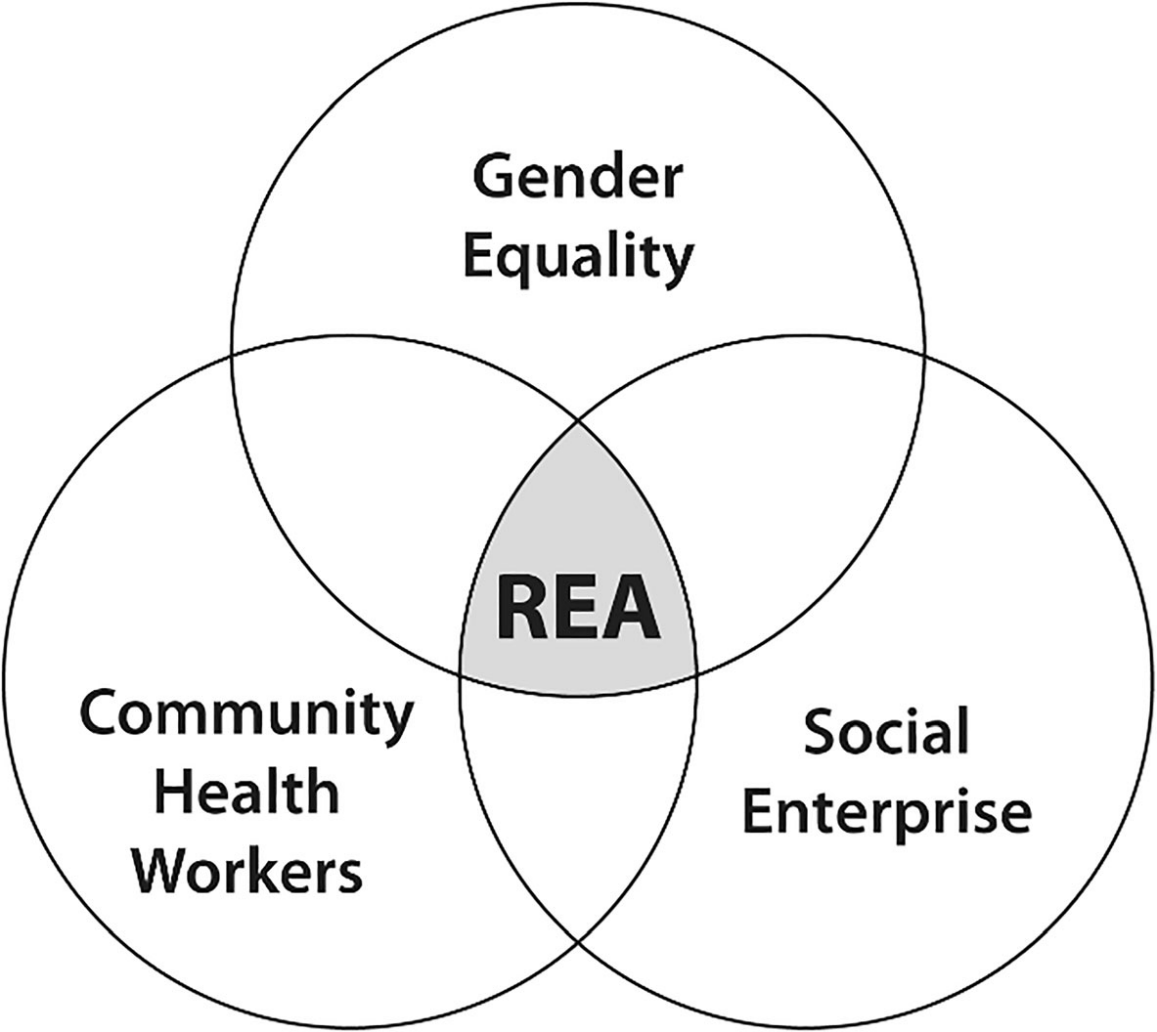
Rapid evidence assessment at the intersection of three fields of knowledge

Working with a Venn diagram representing our three overlapping knowledge domains of interest, we conducted a REA at the intersection of gender equality, social enterprise, and CHWs, with a particular focus on MCH in Africa (see Fig. 1). To our knowledge, this is the first assessment of its kind that seeks to understand the key questions at the intersection of these three domains of knowledge.

A REA is a systematic method for identifying and evaluating existing empirical research [7] and is particularly suited for reviews at the intersection of overlapping disciplines. This method is common in healthcare and is increasingly used to assess emerging issues in management [1, 40]. The REA methodology is designed to identify emerging knowledge more quickly and with less dedication of resources than an exhaustive systematic literature review. The timely findings from a REA are therefore consistent with the needs of practitioners, entrepreneurs, social investors, and policy makers, who often need to make more immediate decisions based on current information. In order to be rapid and cover multiple intersecting areas of knowledge, a REA is not designed to be comprehensive review of every study [1]. Instead, it is designed to be a cost- and resource-efficient review that allows high-level findings to be generated in a timely manner. Our overarching research question was:

*What are the most important research questions about the influence of gender on CHWs working with health social enterprises in Africa?*

With this question as a guide, the assessment began by defining criteria for including articles and reports. The inclusion criteria were that articles had to be in English and published between 1997 and 2017. We included qualitative and quantitative papers in academic journals, as well as practitioner reports in the grey literature. Priority was given to studies based in Africa; low-income, middle-income, or resource-limited regions outside of Africa were included secondarily. We sought to include studies on gender and CHWs in a social enterprise context; however, none were identified, so research on CHWs in both non-profit and government settings were included. Studies that focused on high-income countries and/or focused on workers who are directly part of a formal hospital or facility-based centre (e.g. nurses, midwives, and other auxiliary workers) were excluded.

### Search strategy

Early in the process the REA included correspondence and interviews with experts in the area to identify key studies that should be included. We then proceeded to conduct our search for existing knowledge using Google Scholar. The Google Scholar algorithm is well-suited to exploring overlapping disciplines and gathering findings from both the academic and grey literatures (e.g., reports, working papers, and conference papers), as it returns results based on relevancy of source citations [38]. Although a debate in the literature exists [3, 14], a recent study has found that Google Scholar is 100% effective even for use in gold standard systematic literature reviews [8].

### Identification of studies for full-text review

**Fig. 2:**
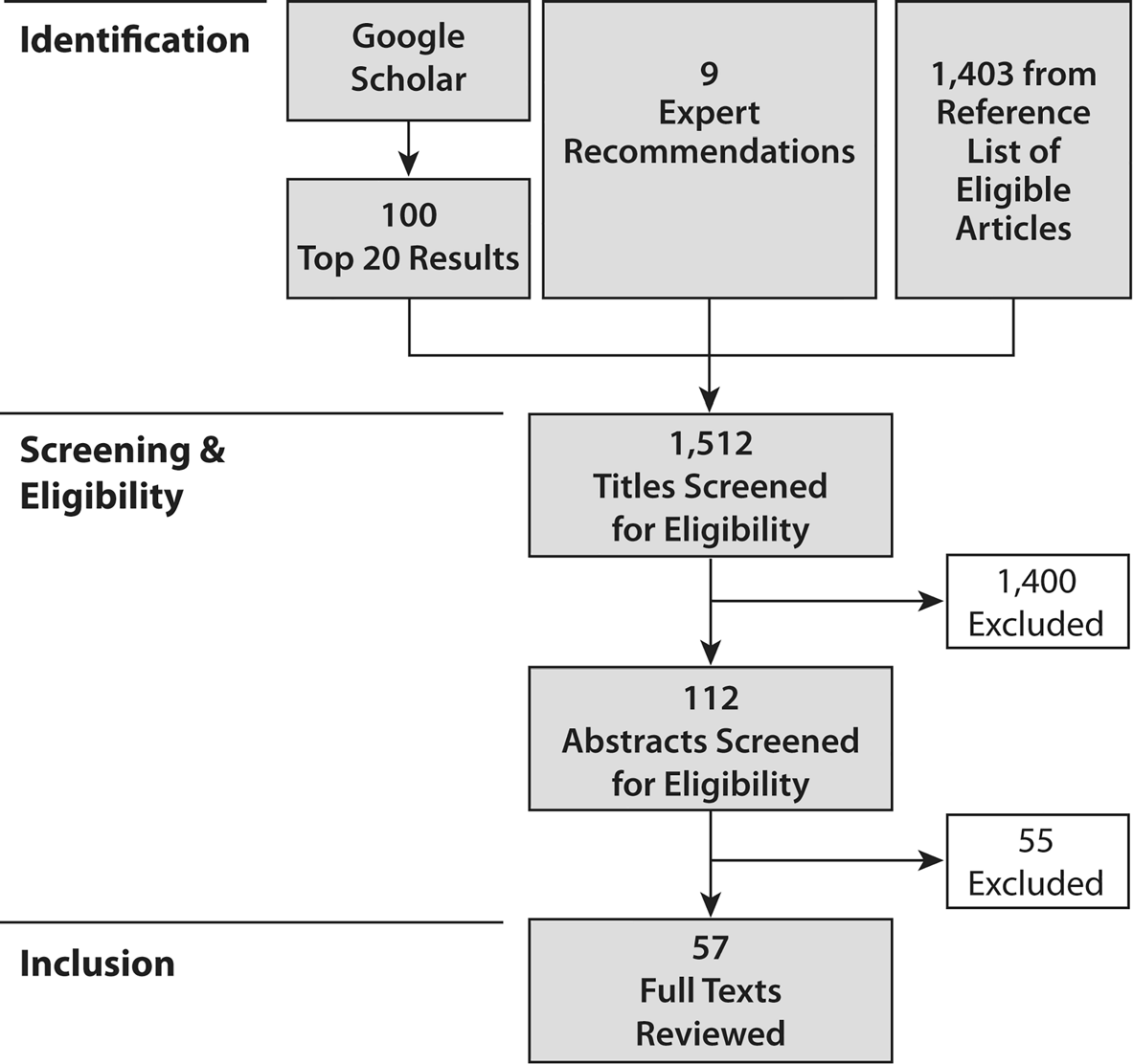
Rapid evidence assessment identification, screening and inclusion

After the initial identification of titles, studies were selected in two phases: selection of titles for abstract review and selection of abstracts for full-text review (see Fig. 2). Five searches were used in Google Scholar, using combinations of the primary search terms: ‘gender’, ‘social enterprise’, and ‘community health worker’, as well as ‘maternal and child health’. The top 20 relevant results per search went on to abstract review. If there was any doubt as to whether a study should be in the top 20, it was included for further full-text review.

After consulting with experts in the fields of interest, nine articles were flagged and three went on to abstract and full-text review. The first Google Scholar search explored the terms ‘gender’ and ‘community health worker’. Google Scholar returned 7560 results. The top 20 abstracts were screened against the inclusion criteria. This yielded four studies for abstract review. After examination, two studies went on for review of the full text. For the second Google Scholar search, we explored the terms ‘gender’ and ‘social enterprise’, which yielded 15,700 results. The top 20 abstracts were screened against the inclusion criteria. This yielded five studies for abstract review. However, none of the five had an emphasis on CHWs, MCH, or Africa.

The third Google Scholar search explored the terms ‘social enterprise’ and ‘community health worker’, which yielded 97 results. The top 20 results were reviewed. These abstracts, however, had a distinct focus on technology and most of the results referred to social enterprises that were involved as technology providers. Some of these studies could have been helpful to our focus had the studies disaggregated their data by sex. However, this was not the case, and though seven studies were highlighted for abstract review, only one study was selected for full-text review.

The terms for the fourth Google Scholar search combined all three central terms of our assessment: ‘gender’, ‘social enterprise’, and ‘community health worker’. This search yielded 57 results. The top 20 results were reviewed. Two studies were selected for abstract review and subsequently, one was kept for full-text review. The fifth search on Google Scholar added a screen for maternal and child health. It searched for the terms ‘gender’, ‘community health worker’, and ‘maternal and child health’, which returned 1940 results. The top 20 results were reviewed and 11 were selected for abstract review, of which eight were kept for full text review.

In addition, we reviewed the reference lists of the eligible articles, which resulted in an additional 1403 titles being identified (of which 80 titles went on to abstract review and 42 abstracts went on to full-text review). In total, 1512 titles were identified, which resulted in 112 abstracts being reviewed. Of these, 57 articles were included for full-text review and coding.

### Full-text review

The final 57 articles were read and thematically coded by hand during the search, selection, and review process [28] to generate a draft list of key research questions. Questions were refined and categorized until the questions and themes remained stable and the best fit with the evidence was found.

## Results

**Fig. 3:**
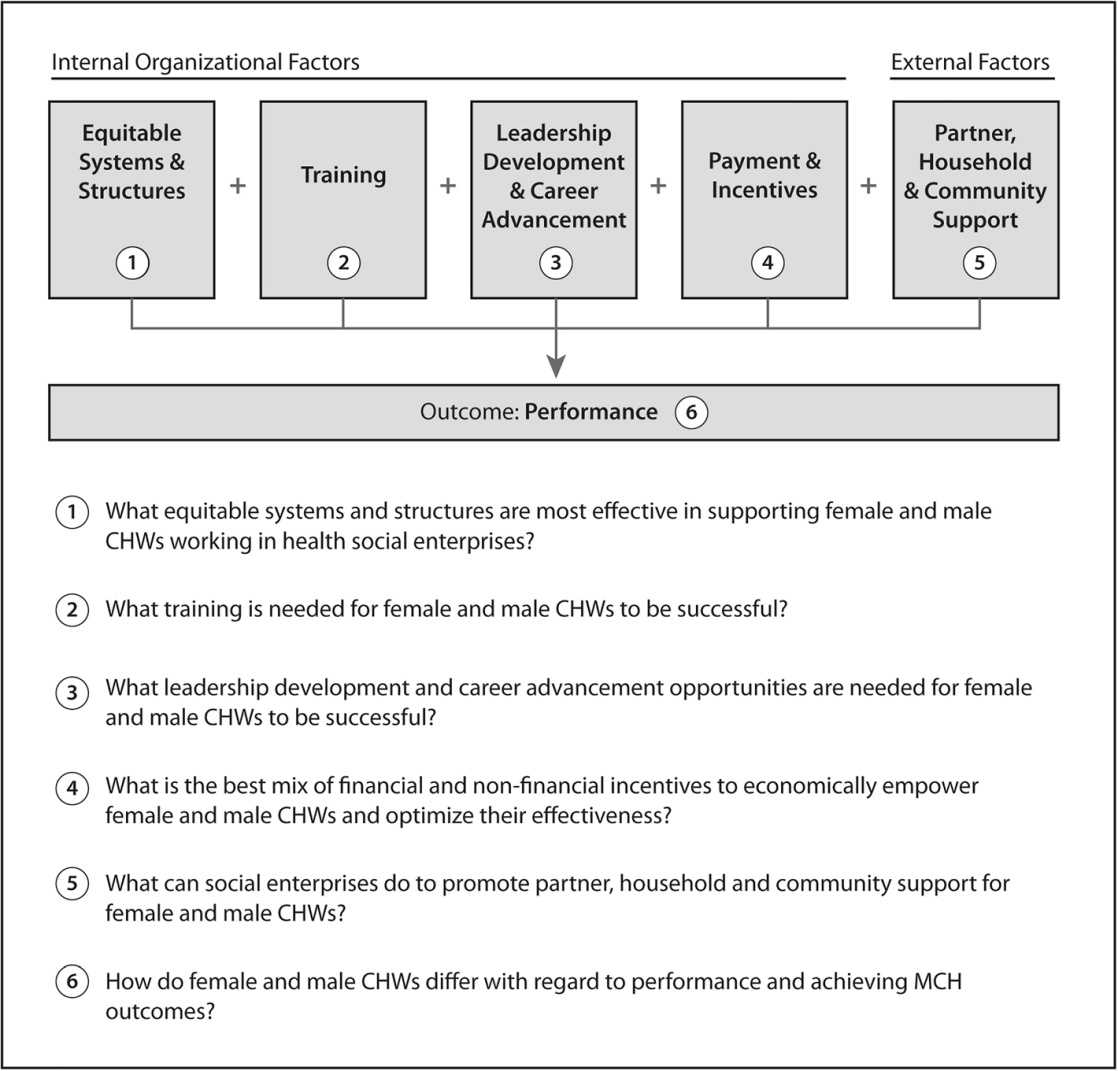
Research agenda with key future research questions

Key research themes and questions emerged in terms of internal organizational factors (equitable systems and structure; training; leadership development and career advancement; payment and incentives), external factors (partner, household, and community support) and performance outcomes (see Fig. 3). Each factor and its associated research question is discussed further below. Once the six key research themes and questions had emerged from the data, we wondered how they related to each other. After reviewing the six emergent questions, we found that they could be further organized into the framework presented in Fig. 3.

### Equitable systems and structures

The success of a CHW program relies on an organization’s policies as well as quality supervision and organizational support systems [24]. Similarly, a social enterprises’ systems are also critical in shaping employees’ engagement and experiences [10]. Forms of equitable systems and structures include employment equity and non-discrimination policies, but also reliable provision of basic toolkits, drug supplies and equipment, without which CHWs cannot do their job effectively [2, 15, 24]. Based on CHW’s complaints, supervisor’s lack of skills, time, and transportation are the primary factors affecting the implementation of MCH programs [11]. In Pakistan, it was found that 70 % of female CHWs reported the most common problem they faced was dealing with administrative inefficiencies, such as inconsistent medical supplies and irregular supply of vaccines; it was also one of the main factors contributing to occupational stress and job dissatisfaction [16].

Although having timely and high-quality supplies is important for both women and men, many of these supports have additional gender dimensions and are further complicated for women due to the socially constructed expectations related to their roles. For instance, drug supplies may be unreliable for everyone, but women are often further burdened by limited mobility and may not have access to transport or the required funds needed to purchase supplies. Notably, CHWs with regular access to curative commodities and medicines had a higher social standing than those without [9]. These challenges are compounded by the fact that women must often work harder than men to be accepted by their communities and to overcome negative stereotypes [29].

The literature also reflects the critical importance of CHWs having effective and supportive supervisors, and supportive management systems. However, appropriate supervision is often one of the weakest links in CHW programs as a result of poorly defined roles and the difficulty of providing supervision in remote areas where services are already over-stretched and ill-equipped (Haines et al., 2011; [24]). Additionally, in light of the current emphasis on task-shifting, there is a real danger of overloading CHWs [43]. In Rwanda, the key challenges for CHWs included an overwhelming workload and a lack of sufficient supervision [4]. Therefore, from our assessment, we formulated the following research question needing further study:

*What equitable systems and structures are most effective in supporting female and male CHWs working in health social enterprises?*

### Training

The CHW literature emphasizes the importance of training. Most CHWs receive some training, often a few weeks but in some cases up to six months. As there is no formal professional or para-professional certification, however, the content, quality, length of, responsibility for, and approaches to training CHWs varies between programs and organizations [24]. Related to our interest in health social enterprises, there is also growing evidence that shows businesses are relevant actors in enhancing women’s access to training [10].

Research on training from Pakistan found that female CHWs operate within socially constructed gender norms that disadvantages and marginalizes them relative to the male-dominated society in which they live and work [29]. It is not surprising, then, that related to training issues, female CHWs reported sexual harassment, lack of understanding of women’s limited mobility and other gender-based constraints by their employers as some of their priority concerns [29]. Similarly, it was found that a quarter of female CHWs have significant occupational stress in Pakistan [16]. However, having greater skills and appropriate training including stronger communication reduces such stress [16].

The quality of training is a critical factor in the success of any CHW program, which requires adequate investment [32]. Challenges with training persist where CHWs described training as insufficient, poor quality, irrelevant, and inflexible and requested further training on counselling, communication and topics outside of their role [11]. In Rwanda, irregular trainings were an important constraint faced by CHWs [4]. In addition, depending on the context, women and men will have varying levels of education and literacy and will be operating under different social norms and expectations. However, no current research has been conducted on how female and male’s differing needs could be addressed by varied training. We argue that it is critical to consider these factors when training female and male CHWs, ensuring that training is gender sensitive and responsive. As a result, we formulated the research question,

*What training is needed for female and male CHWs to be successful?*

### Leadership Development and career advancement

Leadership development and career advancement are highlighted throughout the literature as important to consider, particularly for female CHWs. The perceived absence of professional development opportunities and lack of career paths were additional factors associated with occupational stress for female CHWs [16], which led the researchers to recommend that a structured career path should be set out to improve performance [16]. Another study found that the development of career paths for women would aid in gaining respect from male colleagues and improving women’s job satisfaction [29], and subsequently, it was recommended that CHW programs should establish rewards and clear pathways for promotion [29]. Research also shows that some CHWs would appreciate the opportunity to share experiences with fellow CHWs [11].

Research has also recommended reserving a percentage of higher-level management positions for women in order to involve women at all levels of decision-making [29]. These findings are supported by a recent study that explored gender integration within social enterprises, which found that integrating women into middle and senior management is critical but requires long-term investment in capacity building and leadership development for women [10]. Improved engagement of female managers and employees (e.g. at-work training, opportunities in non-traditional roles, etc.) was found to have the potential to increase organizational productivity and performance [10]. As a result of the evidence, our question for further research was formulated as follows:

*What leadership development and career advancement opportunities are needed for female and male CHWs to be successful?*

### Payment and incentives

An on-going and sometimes contentious debate around whether CHWs should be volunteers or paid for their work continues in the literature and in practice [24]. On one hand, volunteer CHWs are seen as a more sustainable, community-based approach to provide frontline MCH care in low-resource settings. Volunteers often receive a small financial incentive, such as an honorarium, travel allowance, sales from the sale of medicines, or other irregular payments [24], but are also motivated by non-financial incentives, such as social recognition and prestige, the opportunity to acquire greater health knowledge, and access to medicines that benefit their families [12]. On the other hand, it is argued that without adequate compensation, because CHWs are often poor, marginalized women, with significant time burdens and responsibilities, some see these volunteer positions as gender-based exploitation [9, 12]; and these organizations may also be subject to challenges such as high turnover and attrition [24].

Related to payment, there is a need for further research that examines CHW motivation by demographic characteristics including gender [13]. One review found that CHWs are motivated by altruism and social recognition, but also by knowledge gain and career development, and moreover, some are demotivated when their services are not appreciated [11]. Some CHWs wanted regular payment, while others worried that payment might threaten their status, and some salaried CHWs were dissatisfied with their pay levels [11]. In Rwanda, performance-based financing was an important incentive, but CHWs were also strongly motivated by community respect [4]. Tanzanian CHWs also have an intrinsic desire to volunteer, but this does not preclude a desire for external rewards [13]; and adequate financial incentives and in-kind alternatives were found to reduce the burden on families and increase a CHW’s commitment [13]. Subsequently, the essential future research question we have formulated reflects this ongoing debate:

*What is the best mix of financial and non-financial incentives to economically empower female and male CHWs and optimize their effectiveness?*

### Partner, household, and community support

The literature notes that external support from partners, households and the community increase effectiveness and sustainability of CHW initiatives [15]. It is seen that gender bias and a lack at support starts at home, where active support from family members has a big impact of CHWs’ experiences [11]. Husband’s resistance and lack of support was a key barrier to female CHW’s participation in Peru [24]. Similarly, the main reason for female CHWs not attending training (and limiting advancement) in India was a lack of support from their partners (a prerequisite for promotion), whereas male CHWs saw support as an entitlement because it would increase their earning potential [9]. To mitigate these impacts, innovative approaches are needed to address partner resistance and lack of household support; for instance, researchers recommend actively engaging male partners and household members to educate them and address concerns [23]. A lack of support can also impact service delivery. Indian female CHWs reported being afraid to walk on their own between villages due to inadequate lighting and harassment, which required them to rely on spouses and other people for support [9].

Gender bias extends beyond the household though and to the communities that CHWs serve, which can exacerbate the existing gender issues around social acceptance and personal security. Consistent community support has been found to be a key component in the success of CHW programs [15]. In Tanzania, female CHWs struggled to provide counselling due to a lack of acceptance during home visits, despite having a similar knowledge base as men, as their motivations were thought to be secret or were misunderstood as adulterous [6]. Meanwhile, male CHWs faced gender bias as well in struggling to be accepted during home visits to pregnant women [6]. The transgression of gender norms is one of the primary factors increasing risks for female CHWs. Female CHWs who break traditional gender norms in Bangladesh can be ridiculed or South African CHW’s para-professional status can threaten the social status of their male partners [9]. CHWs can also be perceived as immoral due to their involvement with delicate subjects such as family planning or because they interact with male colleagues and travel unchaperoned, which in some cases has created backlash and even violence [9]. Based on the evidence in the literature, we propose the following important question for future research:

*What can social enterprises do to promote partner, household and community support for female and male CHWs?*

### Performance

Robust evidence on the effects of CHWs for improving MCH is limited, however their use shows promising benefits when compared to usual care [25]. Different studies included CHWs performing different activities related to improving MCH, including reducing under-nutrition and maternal and child mortality, as well as controlling malaria, TB and HIV/AIDS. The majority of articles reviewed do not disaggregate findings or performance data by sex or gender. This is consistent with the findings of other reviews that have not found specific evidence on the relative effectiveness of female versus male CHWs [15]. In Kenya, where CHWs were observed during pregnancy home visits, it was found that socio-demographic characteristics such as the age, sex and education of CHWs has an impact on performance [5]. Where male CHWs were more likely to keep better records, female CHWs were more likely to counsel their clients appropriately and to elicit behaviour change [5]. Due to these findings, the researchers recommend that female CHWs are best suited to undertake MCH interventions [5]. Supporting this recommendation, in Somalia it was found that male CHWs experienced challenges in providing reproductive counselling and health services [6]. In Nigeria, low acceptance of male CHWs negatively impacted their performance; while in Afghanistan, the presence of a female CHWs was associated with higher utilization of reproductive services [6]. Such findings suggest that female-to-female or male-to-male health delivery services may improve performance and effectiveness.

However, these findings contrast with a study from Western Uganda that assessed the ability of CHWs to assess pneumonia in children under five, which found no relation between sex and performance [5]. Moreover, it has been argued that this perspective can reinforce existing gender bias in maintaining the assumption that only women should be responsible for MCH [6]. These assumptions also do not necessarily change or influence established gender norms, and subsequently inequalities, that may be restrictive or disadvantaging women in the first place. This has been seen in Brazil, where relying on female staff reinforced the assumption that only women can provide MCH advice, which was felt to excuse men from taking responsibility for childcare [9]. In Indonesia, the social status of elite female CHWs in fact supported successful programming but also reinforced stereotypical notions around female domesticity, voluntarism, and caregiving [9]. Researchers have thus recommended that more effort is required, when relying on an all-female workforce, to avoid the entrenching of gender stereotypes [9]. Therefore, based on the evidence available, we propose the following research question:

*How do female and male CHWs differ with regard to performance and achieving MCH outcomes?*

## Discussion

The Sustainable Development Goals have reinforced the importance of gender equality, maternal and child health, and partnerships with the private sector to improve the lives of women and people everywhere. Given the growing interest in gender equality and social enterprises using community health workers in Africa, this rapid evidence assessment reviewed the literature at the intersection of these fields and proposed a research agenda of six important questions for this emerging area of knowledge and practice.

Our assessment has some limitations associated with the REA methodology, as the findings are not necessarily as exhaustive as an extensive systematic literature review. However, this methodology was intentionally selected for its suitability in quickly identifying key findings in an emerging area of knowledge. The REA also significantly relies on the judgment of the researchers to determine inclusion of studies and to identify the most relevant themes. These challenges aside, the REA can be a powerful tool for considering the intersections of distinct fields of knowledge.

## Conclusion

Given the pervasiveness of gender inequality in health systems, social enterprises have an opportunity to better understand the extent to which gender dynamics and inequalities impact their work. Future research to answer the key questions generated by this REA can ultimately contribute to the ability of social enterprises to improve health outcomes for women and their families along with improving greater equality overall and increased economic empowerment for both female and male CHWs.

## Data Availability

This rapid evidence assessment reviewed publically available publications.

## Abbreviations

CHWs: Community Health Workers
REA: Rapid Evidence Assessment
SDGs: Sustainable Development Goals
